# Time to epinephrine treatment is associated with the risk of mortality in children who achieve sustained ROSC after traumatic out-of-hospital cardiac arrest

**DOI:** 10.1186/s13054-019-2391-z

**Published:** 2019-03-27

**Authors:** Yan-Ren Lin, Meng-Huan Wu, Tren-Yi Chen, Yuan-Jhen Syue, Mei-Chueh Yang, Tsung-Han Lee, Chih-Ming Lin, Chu-Chung Chou, Chin-Fu Chang, Chao-Jui Li

**Affiliations:** 1 Department of Emergency Medicine, Changhua Christian Hospital, Changhua, Taiwan; 2 School of Medicine, Kaohsiung Medical University, Kaohsiung, Taiwan; 3 School of Medicine, Chung Shan Medical University, Taichung, Taiwan; 4 Department of Emergency Medicine, Kaohsiung Chang Gung Memorial Hospital, Chang Gung University College of Medicine, No. 123, Dapi Rd., Niaosong Dist., Kaohsiung, 833 Taiwan; 5 Department of Anaesthesiology, Kaohsiung Chang Gung Memorial Hospital, Chang Gung University College of Medicine, Kaohsiung, Taiwan; 6 Department of Neurology, Changhua Christian Hospital, Changhua, Taiwan; 7 Department of Social Work and Child Welfare, Providence University, Taichung, Taiwan; 8 Department of Medicinal Botanicals and Health Applications, Da-Yeh University, Changhua, Taiwan; 9 Department of Leisure and Sports Management, Cheng Shiu University, Kaohsiung, Taiwan

**Keywords:** Epinephrine, OHCA, Children, Traumatic, Survival

## Abstract

**Background:**

The benefits of early epinephrine administration in pediatric with nontraumatic out-of-hospital cardiac arrest (OHCA) have been reported; however, the effects in pediatric cases of traumatic OHCA are unclear. Since the volume-related pharmacokinetics of early epinephrine may differ obviously with and without hemorrhagic shock (HS), beneficial or harmful effects of nonselective epinephrine stimulation (alpha and beta agonists) may also be enhanced with early administration. In this study, we aimed to analyze the therapeutic effect of early epinephrine administration in pediatric cases of HS and non-HS traumatic OHCA.

**Methods:**

This was a multicenter retrospective study (2003–2014). Children (aged ≤ 19 years) who experienced traumatic OHCA and were administered epinephrine for resuscitation were included. Children were classified into the HS (blood loss > 30% of total body fluid) and non-HS groups. The demographics, outcomes, postresuscitation hemodynamics (the first hour) after the sustained return of spontaneous circulation (ROSC), and survival durations were analyzed and correlated with the time to epinephrine administration (early < 15, middle 15–30, late > 30 min) in the HS and non-HS groups. Cox regression analysis was used to adjust for risk factors of mortality.

**Results:**

A total of 509 children were included. Most of them (*n* = 348, 68.4%) had HS OHCA. Early epinephrine administration was implemented in 131 (25.7%) children. In both the HS and non-HS groups, early epinephrine administration was associated with achieving sustained ROSC (both *p* < 0.05) but was not related to survival or good neurological outcomes (without adjusting for confounding factors). However, early epinephrine administration in the HS group increased cardiac output but induced metabolic acidosis and decreased urine output during the initial postresuscitation period (all *p* < 0.05). After adjusting for confounding factors, early epinephrine administration was a risk factor of mortality in the HS group (HR 4.52, 95% CI 2.73–15.91).

**Conclusion:**

Early epinephrine was significantly associated with achieving sustained ROSC in pediatric cases of HS and non-HS traumatic OHCA. For children with HS, early epinephrine administration was associated with both beneficial (increased cardiac output) and harmful effects (decreased urine output and metabolic acidosis) during the postresuscitation period. More importantly, early epinephrine was a risk factor associated with mortality in the HS group.

## Background

Among adult patients, epinephrine treatment has been demonstrated to increase the likelihood of out-of-hospital cardiac arrest (OHCA) patients achieving the return of spontaneous circulation (ROSC) by increasing the cardiac output, aortic diastolic pressure, cardiac contractility, and coronary blood flow (via binding to alpha-2 or beta-1 receptors) [1–4]. Moreover, some previous studies have even recommended that epinephrine should be administered as quickly as possible (i.e., prehospital loading) once a nonshockable rhythm was identified [3, 5, 6]. Although the benefits of epinephrine for treating adult OHCA patients have been clearly reported, the harmful effects (postresuscitation cardiac dysfunction, arrythmias, tissue microcirculation impairments, and early brain ischemia) that might impair survival or neurological outcomes have also been questioned in some recent large studies [4, 7–11]. Recently, a few pediatric population studies have reported that early epinephrine treatment might be beneficial for treating nontraumatic cardiac arrest in children [5, 12, 13]. However, among these studies, the subgroup of children with traumatic OHCA was not well identified and analyzed.

Clinically, traumatic cardiac arrest can be mainly attributed to hemorrhagic shock (HS)- or non-hemorrhagic shock (non-HS)-related causes (e.g., head and neck injury or respiratory tract injury) [14, 15]. Although the management of these patients obviously differs depending on the injuries (e.g., airway protection for tracheal injury, volume resuscitation, and hemorrhage control for massive hemorrhage), the strategies for administering epinephrine during cardiopulmonary resuscitation (CPR) have not been totally replaced in either HS or non-HS patients. Some pharmacological studies have demonstrated that drug effects could be enhanced under conditions of HS [16–18]. Early treatment with epinephrine or vasopressin can cause tissue ischemia and even lead to a poor prognosis, especially in those receiving epinephrine before appropriate fluid resuscitation [19–21]. Therefore, we suspect that early epinephrine treatment might increase the likelihood of harmful effects in children with HS OHCA. Unfortunately, this hypothesis has not been verified. In this study, we aimed to analyze the therapeutic effect of early epinephrine administration in pediatric cases of HS and non-HS traumatic OHCA.

## Methods

### Study design

This was a multicenter, pre/in-hospital medical record review study. During the study period (January 1, 2003, to December 31, 2014), children who suffered from traumatic OHCA and received epinephrine for resuscitation were analyzed. The time to epinephrine treatment, patient characteristics, and major mechanism of arrest (HS or non-HS) were analyzed retrospectively and correlated with outcomes.

### Ethics statement

This study was performed with the permission of the institutional review board (IRB) of one medical center in central Taiwan. All review work was performed by emergency department (ED) physicians using a standardized abstraction form. The quality of the review was monitored by regular meetings, and the final output data were deidentified.

### Study setting and population

#### Inclusion and exclusion criteria

**Fig. 1:**
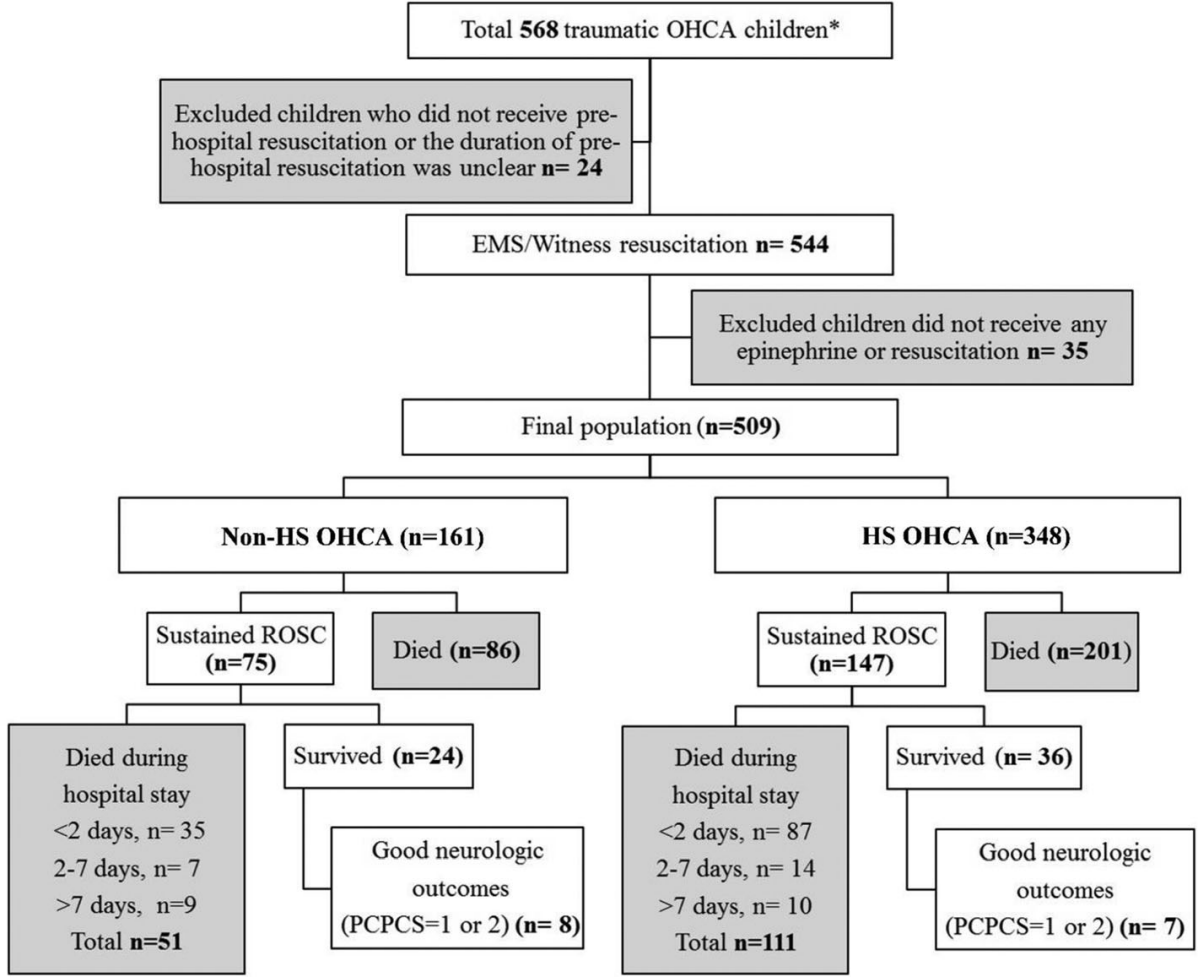
Selection principles and primary outcomes of children with traumatic OHCA. *Age ≤ 19 years

The inclusion principles are shown in Fig. 1. During the study period, a total of 568 children with traumatic OHCA (age ≤ 19 years) admitted to the EDs of three medical centers were included. Trauma mechanisms that might be associated with intoxication, drowning, or burn injury were not included. Because this study focused on the time to epinephrine treatment, children who did not receive prehospital resuscitation or did not have the duration of resuscitation recorded were excluded. Moreover, 35 children who did not receive any epinephrine or resuscitation treatment were excluded. Among these 35 patients, 29 were declared dead by ED physicists on arrival (with characteristics of rigor mortis, livor mortis, or vital organ exposure). The other six patients achieved ROSC in a very short time and did not need any epinephrine. Because this study focused on analyzing the effect of epinephrine and the patient number was very small, we do not discuss these six patients in this study. The final study population included 509 children.

#### Emergency medical service (EMS) and hospital facilities

The three medical centers were located in northern (3700 beds), central (2500 beds), and southern (2500 beds) Taiwan. These centers covered a population of 2,600,000 pediatric individuals, and electronic medical records were shared. The ED physicians of these hospitals were emergency medicine specialists. Before arriving at the hospital, patients were resuscitated by EMS personnel at the scene or in the ambulance. In cases of traumatic OHCA, the EMS personnel consistently followed the “traumatic OHCA” protocol (supervised by EMS medical directors). They performed basic life support (BLS), airway management (using a laryngeal mask airway, as per routine), an automated external defibrillation (AED) assessment, bleeding control (direct pressure, splinting of fractures and the use of tourniquets), fluid administration (20 mL/kg for children or 1 L for adults, as possible), and medication administration. During the study period, the median EMS response time was 6 min (interquartile range (IQR) 4 min), and the median transportation time was 12 min (IQR 6 min). The resuscitation techniques were performed in accordance with the law. Additionally, the quality of resuscitation was regularly monitored by the EMS medical directors. In the ED, patients were treated according to the standard advanced pediatric life support (APLS), advanced trauma life support (ATLS), or advanced cardiovascular life support (ACLS) protocol. In addition, the dose of epinephrine was determined according to the body weight of each patient. The decision to terminate resuscitation was made only by ED physicians when CPR lasted longer than 1 h without any ROSC.

### Study protocol

#### Prehospitalization information and patient characteristics

Prehospitalization information of the 509 children was obtained from their prehospitalization EMS records and in-hospital medical records. Patient characteristics, including age (infant < 1 year, toddler 1–4 years, preschool 5–9 years, school-age 10–14 years, adolescent 15–19 years), sex, major site of injury (head and neck, thorax, abdomen, multiple regions), mechanism of injury (motor vehicle crash/road traffic injury, fall, crush injury, others), and type of trauma (blunt or penetrating) were obtained. Multiple trauma was defined as more than one site of trauma. Prehospitalization information, including the time of collapse, duration of time from the scene to the hospital, and duration of prehospitalization BLS, was recorded.

#### ED resuscitation and time to epinephrine treatment

The ED resuscitation data included the initial cardiac rhythm (on arrival) and in-hospital CPR duration. The initial cardiac rhythm was classified as ventricular fibrillation (VF), pulseless electrical activity (PEA), or asystole. In this study, VF included pulseless ventricular tachycardia. Children were classified as receiving early tranexamic acid treatment if they were administered tranexamic acid within 60 min (from collapse) [22, 23]. In addition, children were classified as achieving initial volume resuscitation if they were administered a total of 20 mL/kg of fluid (including blood products) for resuscitation (any time from prehospitalization to during ED resuscitation) [24]. For example, if a 20-kg child received a total volume of fluid less than 400 mL before leaving the ED, this child was classified as not achieving initial volume resuscitation. Finally, the year of ED admission was recorded (< 2006, 2006–2010, 2011–2015).

Information regarding the time to epinephrine treatment (early < 15 min, middle 15–30 min, late > 30 min) and the dosage of epinephrine were obtained. The time to epinephrine treatment was defined as the time between collapse and administration of the first dose of epinephrine (administered by EMS personnel or ED physicians).

#### Classification as HS or non-HS

The major mechanism of arrest was classified as HS or non-HS. Patients were included in the HS group if they presented definitive evidence of HS (blood loss > 30% of the total blood volume adjusted to the body weight or age of the patient, ATLS class III or IV hemorrhage) before or during resuscitation [24–26]. The remaining patients were included in the non-HS group. The evidence of HS was evaluated according to the ED or surgical diagnosis. Moreover, the amount of blood loss was evaluated according to the EMS reports, physician’s findings, blood loss/transfusion records, imaging reports (X-ray, computed tomography, or sonography results as reported by radiologists), and surgical findings. Measurements of the volume of hemorrhage from images were obtained according to published information [27–31]. For example, if traumatic intracranial hemorrhage, tension pneumothorax, cardiac tamponade, spinal injury, or airway injury (blood loss not more than 30% of the total blood volume) was the major reason for cardiac arrest, the patients were classified as having HS.

#### Primary outcomes

The primary outcomes were measured according to the pediatric Utstein reporting system and included the achievement of sustained ROSC, survival to discharge, and good neurological outcomes [32, 33]. In this study, sustained ROSC was defined as ROSC lasting longer than 20 min. The neurological outcomes were evaluated with the Pediatric Cerebral Performance Category Scale (PCPCS) at the time of hospital discharge [32, 34]. In this study, good neurological outcomes were defined as PCPCS scores of 1 or 2.

#### Secondary outcomes

The secondary outcomes were the duration of survival (from ED admission to death or hospital discharge) and initial postresuscitation hemodynamic status (during the first hour after achieving sustained ROSC). The initial postresuscitation hemodynamics were chosen and measured according to published information and included the consciousness level (Glasgow Coma Scale (GCS) score > 7, 4–7, or 3; the highest score before therapeutic sedation was selected), cardiac rhythm (sinus, nonsinus) and rate (tachycardia, normal, or bradycardia; the major rhythm and rate were chosen and adjusted to the patient age), mean blood pressure (hypertension, normal, or hypotension; the most predominant blood pressure before the use of vasoactive medications was chosen), urine output (> 1 mL/kg/h, 1–0.5 mL/kg/h, or < 0.5 mL/kg/h; collected using urinary catheters; residual urine was not included), and the presence of metabolic acidosis (blood pH < 7.35) [35, 36]. The oxygenation condition of each patient (hypoxia or normoxia) was evaluated after initial respiratory support (i.e., bag valve mask, ventilation) was established in the postresuscitation period. Patients were sent to the operating room or intensive care unit after achieving ROSC. The mean ED stay during the study period was 60 min.

#### Data analysis

For the descriptive analysis, the chi-squared test, Mann-Whitney *U* test, logistic regression, Cox regression analysis, and Kaplan-Meier curves were used (SPSS statistical package for Windows, Version 15.0, SPSS, Inc., Chicago, IL). Differences in nonnormally distributed demographic data between the HS and non-HS groups are reported as the number and percentage or the median and IQR and were analyzed using the chi-squared or Mann-Whitney *U* test. Furthermore, associations among the primary outcomes (sustained ROSC, survival to discharge, good neurological outcomes), secondary outcomes (initial postresuscitation hemodynamic status), and time to epinephrine (early < 15 min, middle 15–30 min, late > 30 min) were analyzed in both the HS and non-HS groups using the chi-squared test. The primary outcomes were further analyzed by logistic regression separately as dependent variables according to the time to epinephrine treatment, HS status, and time to epinephrine treatment × HS status interaction as predictors. In addition, survival bias might have influenced the results. We also performed an analysis after adjusting for confounding factors of the risk of mortality (Cox regression analysis). These factors included patient characteristics (age, sex), pre/in-hospital information (major site of injury, mechanism of injury, type of trauma, initial cardiac rhythm, period from the scene to the hospital, early tranexamic acid administration, achievement of initial volume resuscitation, the year of ED admission), the postresuscitation hemodynamic status, the time to epinephrine treatment, and interaction terms (time to epinephrine treatment × potential effective factors, including the period from the scene to the hospital, achievement of initial volume resuscitation, early tranexamic acid administration, consciousness level, and urine output). In addition, a Kaplan-Meier survival analysis was performed according to the time to epinephrine treatment in all patients, HS and non-HS patients, and HS and non-HS patients who achieved sustained ROSC. We also performed a retrospective power calculation (post hoc) of the sample size (for survival events in the HS group, *n* = 36) (G*Power V3.1 for Windows). A *p* value < 0.05 was considered statistically significant.

## Results

### Differences between HS and non-HS OHCA

The demographics are shown in Table 1. Most of the children had HS OHCA (*n* = 348, 68.4%). The main sites of trauma differed significantly between the two groups. Multiple locations and the head and neck were the most common sites of trauma in the HS and non-HS groups, respectively. In addition, an initial PEA rhythm was more common in the HS group. Most patients (*n* = 299, 58.7%) received the first dose of epinephrine in the middle time frame (10–15 min). The result of the power calculation for our sample size was 0.91 (*α* error 0.05, effect size *w* 0.60, degrees of freedom 2).

**Table 1:** Demographics of patients with HS and non-HS OHCA

Patient characteristics	Total OHCA (*n* = 509)	HS OHCA (*n* = 348)		Non-HS OHCA (*n* = 161)		
	No. (%)	No.	(%)	No.	(%)	*p* value
Age group^a^						0.002
Infant	24 (4.7)	10	2.9	14	8.7	
Toddler	69 (13.6)	40	11.5	29	18.0	
Preschool	98 (19.3)	68	19.5	30	18.6	
School-age	131 (25.7)	88	25.3.	43	26.7	
Adolescent	187 (36.7)	142	40.8	45	28.0	
Sex						0.092
Male	296 (58.2)	195	56.0	101	62.7	
Female	213 (41.8)	153	44.0	60	37.3	
Major site of injury^a^						< 0.001
Head and neck	202 (39.7)	89	25.6	113	70.2	
Thorax	78 (15.3)	48	13.8	30	18.6	
Abdomen	95 (18.7)	92	26.4	3	1.9	
Multiple areas	134 (26.3)	119	34.2	15	9.3	
Mechanism of injury						0.731
MVC or RTI	351 (69.0)	240	69.0	111	68.9	
Fall	74 (14.5)	50	14.4	24	14.9	
Crush injury	44 (8.6)	28	8.0	16	9.9	
Others	40 (7.9)	30	8.6	10	6.2	
Type of trauma						0.090
Blunt trauma	478 (93.9)	323	92.8	155	96.3	
Penetrating trauma	31 (6.1)	25	7.2	6	3.7	
Prehospital resuscitative phase						
Time from the scene to the hospital (15)^b^ (median, IQR) (min)	17 (4)	16 (5)		17 (4)		0.298
Prehospital BLS duration (median, IQR) (min)	9 (5)	8 (4)		9 (4)		0.415
ED resuscitative phase						
Initial cardiac rhythm^a^						
Asystole	273 (53.6)	188	54.0	85	52.8	0.020
PEA	139 (27.3)	104	29.9	35	21.7	
VF^c^	97 (19.1)	56	16.1	41	25.5	
Achievement of initial volume resuscitation (2)^b^	447 (88.2)	307	88.7	140	87.0	0.565
Early tranexamic acid administration (13)^b^	304 (61.3)	206	60.9	98	62.0	0.818
Year of ED admission						
< 2006	124 (24.4)	85	24.4	39	24.2	0.998
2006–2010	215 (42.2)	147	42.2	68	42.2	
2011–2015	170 (33.4)	116	33.4	54	33.6	
In-hospital CPR duration (median, IQR) (min)	26 (8)	24 (5)		25 (6)		0.420
Administration of epinephrine						
Epinephrine injection (median, IQR) (time)	11 (4)	10 (3)		9 (3)		0.323
Time to epinephrine treatment						0.928
Early (< 15 min)	131 (25.7)	88	25.3	43	26.7	
Middle (15–30 min)	299 (58.7)	205	58.9	94	58.4	
Late (> 30 min)	79 (15.5)	55	15.8	24	14.9	

^a^Significant factor

^b^Number of patients with missing information

^c^VF included pulseless VT

*IQR* interquartile range, *HS* hemorrhage shock, *MVC or RTI* motor vehicle crash or road traffic injury

### Early epinephrine treatment increased sustained ROSC

The selection principles and outcomes are shown in Fig. 1. Although sustained ROSC was achieved in 147 (42.4%, HS OHCA) and 75 children (46.6%, non-HS OHCA), most of them still died during the hospital stay. Early epinephrine treatment was significantly associated with achieving sustained ROSC in both the HS (*p* = 0.017) and non-HS (*p* = 0.001) groups. However, early epinephrine treatment was not significantly associated with survival to discharge or good neurological outcomes. The overall survival rate was 10.3% and 14.9% in the HS and non-HS groups, respectively (Table 2). We also found that early epinephrine administration was the only factor significantly associated with sustained ROSC (OR 5.60, 95% CI 1.88–16.75) but not associated with survival to discharge (OR 3.33, 95% CI 0.67–16.70) or good neurological outcomes (OR 2.35, 95% CI 0.25–22.41). The interaction of the time to epinephrine treatment × HS status was not significant (data not shown).

**Table 2:** Primary outcomes of patients administered epinephrine

Outcomes	HS OHCA (*n* = 348)				*p* value	Non-HS OHCA (*n* = 161)				*p* value
	Time to epinephrine treatment					Time to epinephrine treatment				
	Total	Early (*n* = 88)	Middle (*n* = 205)	Late (*n* = 55)		Total	Early (*n* = 43)	Middle (*n* = 94)	Late (*n* = 24)	
	No. (%)	No. (%)	No. (%)	No. (%)		No. (%)	No. (%)	No. (%)	No. (%)	
Sustained ROSC^a^	147 (42.4)	48 (54.5)	81 (39.5)	18 (32.7)	0.017	75 (46.6)	30 (69.8)	38 (40.4)	7 (29.2)	0.001
Survival to discharge	36 (10.3)	9 (10.2)	22 (10.7)	5 (9.1)	0.938	24 (14.9)	10 (23.3)	12 (12.8)	2 (8.3)	0.172
Good neurological outcomes^b^	7 (2.0)	2 (2.3)	4 (2.0)	1 (1.8)	0.978	8 (5.0)	4 (9.3)	3 (3.2)	1 (4.2)	0.305

^a^Significant factor

^b^Pediatric Cerebral Performance Category Scale (PCPCS) score of 1 or 2

*ROSC* return of spontaneous circulation

### Early epinephrine treatment influenced the postresuscitation hemodynamic status

Among patients who achieved sustained ROSC, the early postresuscitation hemodynamic status differed between patients with HS and non-HS OHCA according to the time to epinephrine treatment. These data are shown in Table 3.

**Table 3:** Early postresuscitation hemodynamics associated with the time to epinephrine treatment

	All children with traumatic OHCA and sustained ROSC (*n* = 222)							
	HS OHCA (*n* = 147)				Non-HS OHCA (*n* = 75)			
	Time to epinephrine treatment				Time to epinephrine treatment			
	Early (*n* = 48)	Middle (*n* = 81)	Late (*n* = 18)	*p* value	Early (*n* = 30)	Middle (*n* = 38)	Late (*n* = 7)	*p* value
	No. (%)	No. (%)	No. (%)		No. (%)	No. (%)	No. (%)	
Consciousness level (GCS score)^a^								
> 7	12 (25.0)	17 (21.0)	2 (11.1)	0.018	9 (30.0)	9 (23.7)	1 (14.3)	0.013
7–4	25 (52.1)	25 (30.9)	5 (27.8)		15 (50.0)	11 (28.9)	0 (0)	
3	11 (22.9)	39 (48.1)	11 (61.1)		6 (20.0)	68 (47.4)	6 (85.7)	
Cardiac rhythm								
Nonsinus rhythm	15 (31.2)	16 (19.8)	5 (27.8)	0.321	9 (30.0)	11 (28.9)	2 (28.6)	0.994
Sinus rhythm	33 (68.8)	65 (80.2)	13 (72.2)		21 (70.0)	27 (71.1)	5 (71.4)	
Heart rate^a^								
Tachycardia	26 (54.2)	44 (54.3)	4 (22.2)	0.014	14 (46.7)	18 (47.4)	2 (28.6)	0.632
Normal	16 (33.3)	19 (23.5)	5 (27.8)		11 (36.7)	11 (28.9)	2 (28.6)	
Bradycardia	6 (12.5)	18 (22.2)	9 (50.0)		5 (16.6)	9 (23.7)	3 (42.8)	
Mean blood pressure^a^								
Hypertension	24 (50.0)	26 (32.1)	3 (16.7)	0.015	14 (46.7)	15 (39.5)	2 (28.6)	0.529
Normal	16 (33.3)	24 (29.6)	5 (27.8)		9 (30.0)	11 (28.9)	1 (14.3)	
Hypotension	8 (16.7)	31 (38.3)	10 (55.6)		7 (23.3)	12 (31.6)	4 (57.1)	
Oxygenation								
Hypoxia	11 (22.9)	26 (32.1)	5 (27.8)	0.535	7 (23.3)	11 (28.9)	2 (28.6)	0.867
Nonhypoxia	37 (77.1)	55 (67.9)	13 (72.2)		23 (76.7)	27 (71.1)	5 (71.4)	
Urine output^a^								
< 0.5 mL/kg/h	23 (47.9)	22 (27.2)	11 (61.1)	0.036	8 (26.7)	8 (21.1)	2 (28.6)	0.941
1–0.5 mL/kg/h	16 (33.3)	39 (48.1)	4 (22.2)		12 (40.0)	19 (50.0)	3 (42.8)	
> 1 mL/kg/h	9 (18.8)	20 (24.7)	3 (16.7)		10 (33.3)	11 (28.9)	2 (28.6)	
Metabolic acidosis (5)^b^								
Yes	35 (77.8)	46 (58.2)	15 (83.3)	0.026	16 (53.3)	17 (44.7)	5 (71.4)	0.401
No	10 (22.2)	33 (41.8)	3 (16.7)		14 (46.7)	21 (55.3)	2 (28.6)	

^a^Significant factor

^b^Number of patients with missing information

*GCS* Glasgow Coma Scale

#### HS OHCA (*n* = 147)

Early epinephrine treatment was significantly related to a better initial GCS score. Fewer patients with a GCS score of 3 received early epinephrine treatment (*n* = 11, 22.9%) than middle (*n* = 39, 48.1%) or late (*n* = 11, 61.1%) epinephrine treatment (*p* = 0.018).

In addition, early and middle epinephrine administration were both significantly associated with tachycardia (*p* = 0.014). Hypertension was more predominant in patients who received early epinephrine treatment. However, while all three time periods showed similar proportions of normotensive patients, early administration (33.3%) showed a slightly higher proportion of normotensive patients than middle (29.6%) and late (27.8%) administration (*p* = 0.015). Urine output < 0.5 mL/kg/h (*p* = 0.036) and metabolic acidosis (*p* = 0.026) were associated with early epinephrine treatment (Table 3).

#### Non-HS OHCA (*n* = 75)

Early epinephrine treatment was also associated with a better initial GCS score (*p* = 0.013) (Table 3).

### Early epinephrine treatment was an adjusted risk factor of mortality after HS OHCA (Cox regression analysis)

The adjusted risk factors of mortality during the postresuscitation period are shown in Table 4. A longer period from the scene to the hospital, not achieving initial volume resuscitation, no early tranexamic acid administration, a poor initial GCS score, decreased urine output, and early epinephrine treatment were significant factors associated with mortality among patients with HS OHCA. The interaction terms were not significant (data not shown).

**Table 4:** Cox regression analysis adjusting for potential risk factors of mortality

Variables^c^	Children with traumatic OHCA and sustained ROSC		
	Total (*n* = 222)	HS OHCA (*n* = 147)	Non-HS OHCA (*n* = 75)
	HR (95% CI)	HR (95% CI)	HR (95% CI)
Time from the scene to the hospital (min)	1.03 (1.01–3.23)^a^	1.04 (1.31–1.56)^a^	1.02 (1.52–4.83)^a^
Achievement of initial volume resuscitation			
No^a^	1.96 (1.08–3.72)^a^	2.84 (1.12–3.06)^a^	1.78 (0.60–4.60)
Yes^b^	1.00	1.00	1.00
Early tranexamic acid administration			
No^a^	1.22 (1.04–2.53)^a^	1.47 (1.10–2.39)^a^	1.30 (0.67–3.22)
Yes^b^	1.00	1.00	1.00
Year of ED admission			
2011–2015	0.84 (0.66–3.01)	0.76 (0.43–1.34)	0.88 (0.73–1.55)
2006–2010	0.96 (0.34–4.21)	1.10 (0.57–2.10)	0.93 (0.46–5.33)
< 2006^b^	1.00	1.00	1.00
Consciousness level (GCS score)			
4–7^a^	5.12 (2.53–10.50)^a^	7.62 (2.85–19.87)^a^	7.52 (2.64–29.30)^a^
3^a^	12.50 (5.71–23.38)^a^	15.05 (4.97–45.45)^a^	19.22 (3.47–105.98)^a^
> 7^b^	1.00	1.00	1.00
Cardiac rhythm			
Nonsinus rhythm	0.96 (0.58–1.55)	1.23 (0.67–3.11)	1.13 (0.52–1.73)
Sinus rhythm^b^	1.00	1.00	1.00
Heart rate			
Tachycardia	1.55 (0.93–2.60)	1.42 (0.76–2.81)	1.59 (0.32–4.66)
Bradycardia	2.51 (0.46–4.32)	2.11 (0.78–3.32)	4.01 (0.90–19.09)
Normal^b^	1.00	1.00	1.00
Mean blood pressure			
Hypertension	0.91 (0.74–2.55)	1.25 (0.88–2.48)	0.55 (0.54–1.22)
Hypotension	1.05 (0.64–1.88)	1.07 (0.74–3.01)	0.94 (0.31–2.57)
Normal^b^	1.00	1.00	1.00
Urine output			
< 0.5 mL/kg/h^a^	1.93 (1.45–3.01)^a^	2.90 (2.11–8.11)^a^	1.52 (0.48–3.72)
1–0.5 mL/kg/h^a^	1.70 (1.42–3.89)^a^	1.96 (1.42–3.86)^a^	1.89 (0.76–9.81)
> 1 mL/kg/h^b^	1.00	1.00	1.00
Metabolic acidosis			
Yes	0.74 (0.49–1.13)	0.92 (0.33–1.01)	0.83 (0.55–4.01)
No^b^	1.00	1.00	1.00
Time to epinephrine treatment			
Early^a^	2.86 (1.98–5.47)^a^	4.52 (2.73–15.91)^a^	0.51 (0.10–2.62)
Middle	0.83 (0.45–1.54)	0.60 (0.26–1.34)	0.39 (0.76–1.94)
Late^b^	1.00	1.00	1.00

^a^Significant factor

^b^Reference group

^c^All variables were also adjusted according to patient characteristics (age, sex), pre/in-hospital information (major site of injury, mechanism of injury, type of trauma, initial cardiac rhythm), and interaction terms (time to epinephrine treatment × potential effective factor, including time from the scene to the hospital, achievement of initial volume resuscitation, early tranexamic acid administration, consciousness level, and urine output)

### Survival curves for different times to epinephrine treatment

**Fig. 2:**
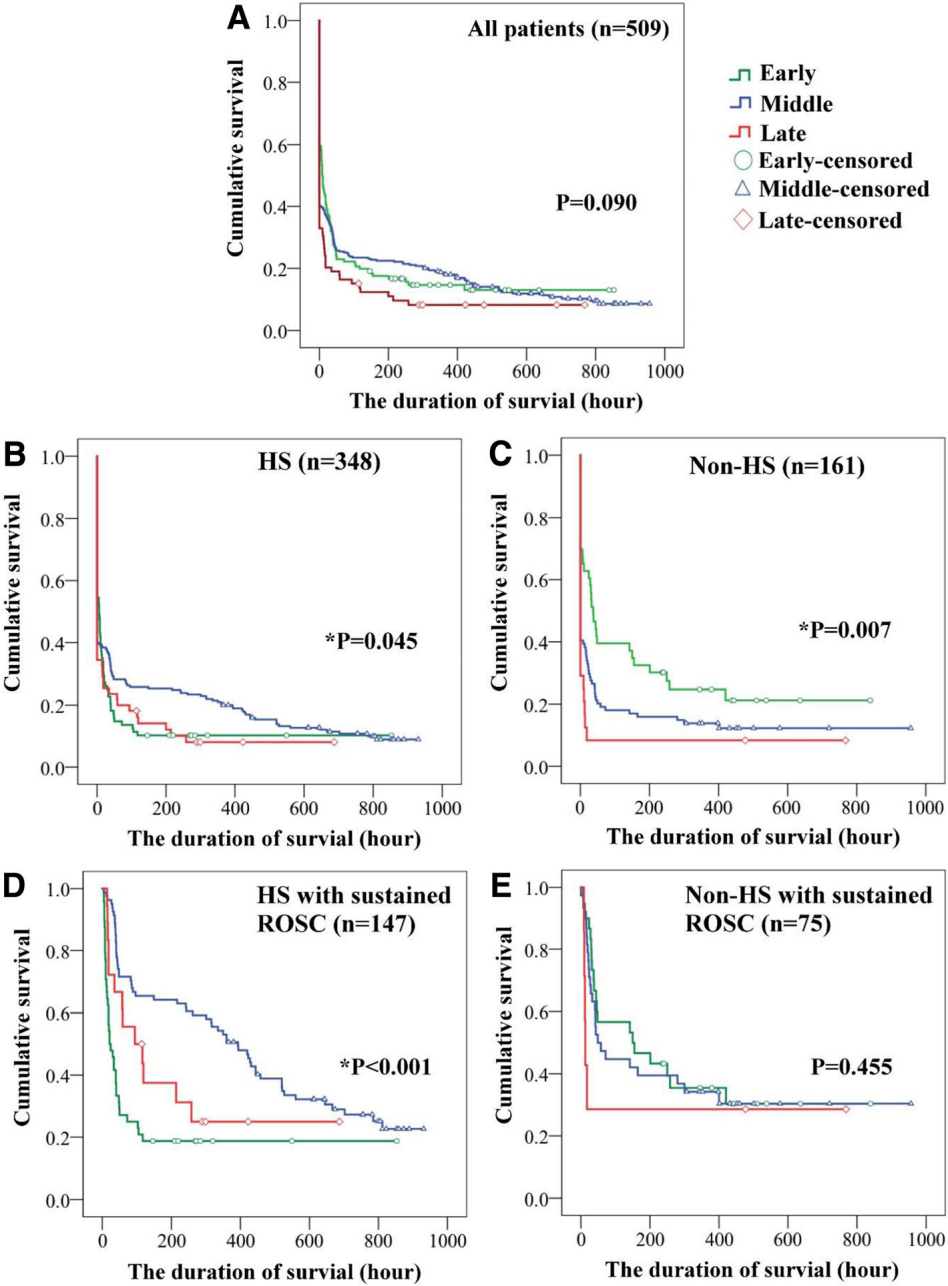
Survival durations of patients with HS and non-HS according to the time to epinephrine treatment. (**a**) All patient, (**b**) patients with HS, (**c**) patients with non-HS, (**d**), HS patients with sustained ROSC, (**e**) non-HS patients with sustained ROSC. For patients with HS, epinephrine administration during the middle stage was associated with a longer survival duration than epinephrine administration during the early or late stage (*p* = 0.045), especially in those who achieved sustained ROSC (*p* < 0.001)

The associations between the survival duration and the time to epinephrine treatment are shown in Fig. 2. The time to epinephrine treatment was not significantly associated with the duration of survival in non-HS OHCA patients with sustained ROSC. However, among patients with HS, treatment with epinephrine in the middle time frame was associated with a longer survival duration than treatment with epinephrine in the early or late time frames (*p* = 0.045), especially in those who achieved sustained ROSC (*p* < 0.001).

## Discussion

Although the beneficial and harmful effects of epinephrine for treating OHCA have been determined in adult patients, this information has not been well addressed in children with traumatic OHCA [4, 10, 37, 38]. In this study, we aimed to analyze the therapeutic effect of early epinephrine treatment in children with HS and non-HS traumatic OHCA.

### General and transient beneficial effects of early epinephrine treatment

In both the HS and non-HS OHCA groups, early epinephrine treatment was not only associated with achieving sustained ROSC but was also related to the subsequent consciousness level (compared with middle or late epinephrine treatment). However, the beneficial effects on brain function were transient because we found that early administration ultimately was not associated with survival to discharge or good neurological outcomes. Although some studies have reported that beta-1 cardiac receptor stimulation by epinephrine increases cardiac output and initially increases brain perfusion, epinephrine ultimately decreases the cortical microcirculation (via alpha-1 and alpha-2 vasoconstriction) [10, 21, 39]. We suspect that once the cardiac output or body fluid volume is decreased (i.e., with persistent hemorrhage), additional brain perfusion is difficult to maintain and ultimately worsens the brain microcirculation. Therefore, we suspect that early epinephrine administration should not be considered the first step in resuscitating children with traumatic OHCA due to major hemorrhage; hemostasis, volume resuscitation, and high-quality CPR might be more important.

### Powerful effect on the cardiovascular system and kidneys in HS OHCA

Early epinephrine treatment was more associated with the cardiovascular system (including more instances of tachycardia and higher mean blood pressures) in children with HS OHCA than in those with non-HS OHCA. Some studies have reported that a higher cardiac output in major trauma patients might worsen bleeding [40–42]. Therefore, for children with HS OHCA, this effect may be more life-threatening, and it is difficult to determine the benefit. Postresuscitation kidney complications (including acute kidney injury, hyperlactatemia, and acidosis) are strong predictors of poor outcomes [43–45]. However, the impact of early epinephrine treatment on postresuscitation kidney function remains unclear. In this study, we found that early epinephrine treatment was obviously associated with harmful effects in terms of the fluid balance and tissue metabolism in patients with HS OHCA. We suspect that the harmful effects of early epinephrine treatment are enhanced in children with HS OHCA.

### Early epinephrine treatment was a risk factor of mortality in children who achieved sustained ROSC after HS OHCA

For patients with HS OHCA, early epinephrine might have harmful effects during the postresuscitation period. In this study, Cox regression analysis was used to adjust for the effects of several variables on the time from a specified event to the time of death. After adjusting for confounding factors, early epinephrine treatment was not the only risk factor of mortality. A longer period from the scene to the hospital, not achieving initial volume resuscitation, no early tranexamic acid administration, a low initial urine output (< 0.5 mL/kg/h), and a GCS score of 3 were also powerful risk factors of mortality. Compared to epinephrine administered during the middle or late stage, epinephrine administration during the early stage was significantly associated with a longer duration of survival in the non-HS group than in the HS group. Therefore, the initial classification of HS or non-HS is important when considering the early administration of epinephrine in children with traumatic OHCA.

## Conclusions

Early epinephrine administration was significantly associated with achieving sustained ROSC in both children with HS and non-HS traumatic OHCA. For children with HS, early epinephrine administration was associated with both beneficial (increased cardiac output) and harmful effects (decreased urine output and metabolic acidosis) during the postresuscitation period. More importantly, early epinephrine treatment was a risk factor associated with mortality in the HS group.

### Limitations

Determining the amount of prehospitalization hemorrhage was the major limitation of this study. Prehospitalization hemorrhage could potentially be underestimated, especially when wounds are not actively bleeding in the ED. A few children with large nonactively bleeding wounds who were not classified as having had a massive bleeding event in EMS records were included in the non-HS group. This was a retrospective study; therefore, it is possible that the time from collapse to the first dose of epinephrine might have been recorded inaccurately. In addition, survival bias might have influenced the results. The association of early epinephrine administration with mortality could be explained as early survival bias. Patients who received epinephrine could have received epinephrine because the likelihood of a positive outcome was estimated to be higher. Early survival did not guarantee long-term survival and could also be a survival bias. Moreover, some other confounding factors associated with survival might not have been fully considered. For example, achieving initial volume resuscitation and early tranexamic acid administration were very important. The time to epinephrine treatment was not the only factor influencing outcomes in the HS group. Early epinephrine administration is only a small piece of the complex puzzle. Extracorporeal membrane oxygenation (ECMO) and hypothermia were not discussed in this study because these are not routine treatments for children with traumatic OHCA [46, 47]. The association between the time to epinephrine treatment and neurological outcomes needs further investigation. There were very few survivors with good neurological outcomes in this study. Large randomized controlled trials should be performed in the future.
